# Enhancement of Photosynthetic Capacity in Spongy Mesophyll Cells in White Leaves of *Actinidia kolomikta*

**DOI:** 10.3389/fpls.2022.856732

**Published:** 2022-05-11

**Authors:** Miao Yu, Li Chen, Dong-huan Liu, Dan Sun, Guang-li Shi, Yan Yin, De-quan Wen, Zhen-xing Wang, Jun Ai

**Affiliations:** ^1^Laboratory of Wild Fruit Physiology, College of Horticulture, Jilin Agricultural University, Changchun, China; ^2^Beijing Botanical Garden, Beijing, China; ^3^Key Laboratory of Plant Resources, State Key Laboratory of Systematic and Envolutionary Botany, State Key Laboratory of Vegetation and Environmental Change, Institute of Botany, Chinese Academy of Sciences, Beijing, China

**Keywords:** white leaf, photosynthetic capacity, leaf structure, chlorophyll fluorescence, spongy mesophyll cells

## Abstract

Considering that *Actinidia kolomikta* bears abundant white leaves on reproductive branches during blossoming, we hypothesized that the white leaves may maintain photosynthetic capacity by adjustments of leaf anatomy and physiological regulation. To test this hypothesis, leaf anatomy, gas exchange, chlorophyll *a* fluorescence, and the transcriptome were examined in white leaves of *A. kolomikta* during flowering. The palisade and spongy mesophyll in the white leaves were thicker than those in green ones. Chloroplast development in palisade parenchyma of white leaves was abnormal, whereas spongy parenchyma of white leaves contained functional chloroplasts. The highest photosynthetic rate of white leaves was ~82% of that of green leaves over the course of the day. In addition, the maximum quantum yield of PSII (*F*_v_/*F*_m_) of the palisade mesophyll in white leaves was significantly lower than those of green ones, whereas *F*_v_/*F*_m_ and quantum yield for electron transport were significantly higher in the spongy mesophyll of white leaves. Photosynthetic capacity regulation of white leaf also was attributed to upregulation or downregulation of some key genes involving in photosynthesis. Particularly, upregulation of sucrose phosphate synthase (*SPS*), glyeraldehyde-3-phosphate dehydrogenase (*GAPDH*) and RuBisCO activase (*RCA*) in white leaf suggested that they might be involved in regulation of sugar synthesis and Rubisco activase in maintaining photosynthetic capacity of white leaf. Conclusions: white leaves contained a thicker mesophyll layer and higher photosynthetic activity in spongy parenchyma cells than those of palisade parenchyma cells. This may compensate for the lowered photosynthetic capacity of the palisade mesophyll. Consequently, white leaves maintain a relatively high photosynthetic capacity in the field.

## Introduction

In nature, the majority of plant leaves are green. Chlorophyll can absorb violet-blue and orange-red light and converts light energy into chemical energy. However, certain plants develop white leaves. Previous studies have shown that white leaves or bracts may serve as “advertisements” to attract pollinators (Williams, 1972; Herrera, 1997; Dufay et al., 2003; Sun et al., 2008; Vekemans et al., 2012; Gagliardi et al., 2016; Song et al., 2018). Generally, it is considered that the specialized function of white leaves may enhance plant reproductive fitness. However, photosynthesis in white leaves is decreased significantly because of chlorophyll deficiency or lower light absorbance (Motohashi et al., 2003; Sun et al., 2008; Užarević et al., 2011; Song et al., 2018; Zhang et al., 2018). Light-harvesting and photosynthetic capacity in white leaves is reported to decrease significantly only when the chlorophyll content decreases drastically (Rosso et al., 2009). However, little difference in photosynthetic capacity is indicated between white leaves and green leaves of *Arum italicum* and *Begonia formosana* (Rocca et al., 2011; Sheue et al., 2012).

Leaf structural traits govern photosynthetic capacity (Holloway-Phillips, 2019). Parenchyma cells on the adaxial side of dorsiventral leaves are collectively termed the palisade mesophyll, which consists of a well-established layer of packed and elongated cylindrical cells, whereas the spongy mesophyll is located on the abaxial side and consists of rounded or irregularly shaped cells. Compared with the spongy mesophyll, the palisade mesophyll contains abundant chloroplasts and the abundance of photosystem II (PSII) reaction centers, electron transport, and Rubisco enzymes is higher in the chloroplasts of palisade parenchyma than those of spongy parenchyma (Nishio, 2000; Borsuk and Brodersen, 2019). Therefore, light absorption by chlorophyll, energy trapping in the PSII reaction centers, electron transport activity, and carbon fixation capacity in the palisade tissue are higher than those in the spongy mesophyll (Evans and Vogelmann, 2003). In addition, the contents of photosynthetic components increases gradually from the adaxial leaf surface to the interior of the leaf, and then the contents decrease in lower mesophyll cells (Borsuk and Brodersen, 2019). Thus, photosynthetic activity of the mesophyll also changes with the variation in composition of the photosynthetic apparatus (Borsuk and Brodersen, 2019). White leaves of *Ranunculus ficaria* and *Arum italicum* (Konoplyova et al., 2008; Rocca et al., 2011) possessed three or two palisade cell layers, which suggested that the leaf structure was altered. However, the authors considered that the change in leaf structure results in increased efficiency of light capture and electron transfer to a certain extent. Furthermore, light absorbance of leaves only decreased 10% when chlorophyll content decreased 50% (Dima et al., 2006; Lysenko, 2012). Thus, it was speculated that light capture is not only associated with chlorophyll content but also with leaf structure and chlorophyll distribution. Therefore, structural changes in leaves might affect the distribution of photosynthetic components and chlorophyll in certain plants, which effectively maintain light absorbance and photosynthetic capacity. Expression of the genes encoding proteins involved in photosynthetic electron transport and Calvin cycle enzymes is correlated with photosynthetic capacity (Gowik and Westhoff, 2011; Foyer et al., 2012). Some albino and chlorina plants have been screened through transcriptome analysis. Genes related to chlorophyll biosynthesis were significantly downregulated, resulting in lower chlorophyll content and photosynthetic capacity (Zhang et al., 2022). Wang et al. (2020) reported that light-harvesting chlorophyll a/b-binding protein (LHC) was closely linked to aberrant chloroplast development in yellow-leaf tea plants. Under the shade, upregulation of chlorophyll a/b-binding protein genes in yellow leaves played an important role in the recovery of photosynthesis activities (Jiang et al., 2020). Furthermore, some genes regulated RuBisCO activity also involved in carbon assimilation, for example, Chloroplast Nucleoids DNA-binding Protease (*CND41*) and RuBisCO activase (*RCA*). Kato et al. (2005) reported that tobacco CND41 protease was involved in Rubisco degradation. Rubisco activase (*RCA*) is an essential gene, which involved in a process necessary for Rubisco activation and carbon fixation. These studies provide useful information illuminating the mechanism of maintenance or reduction in photosynthetic capacity.

**Figure 1:**
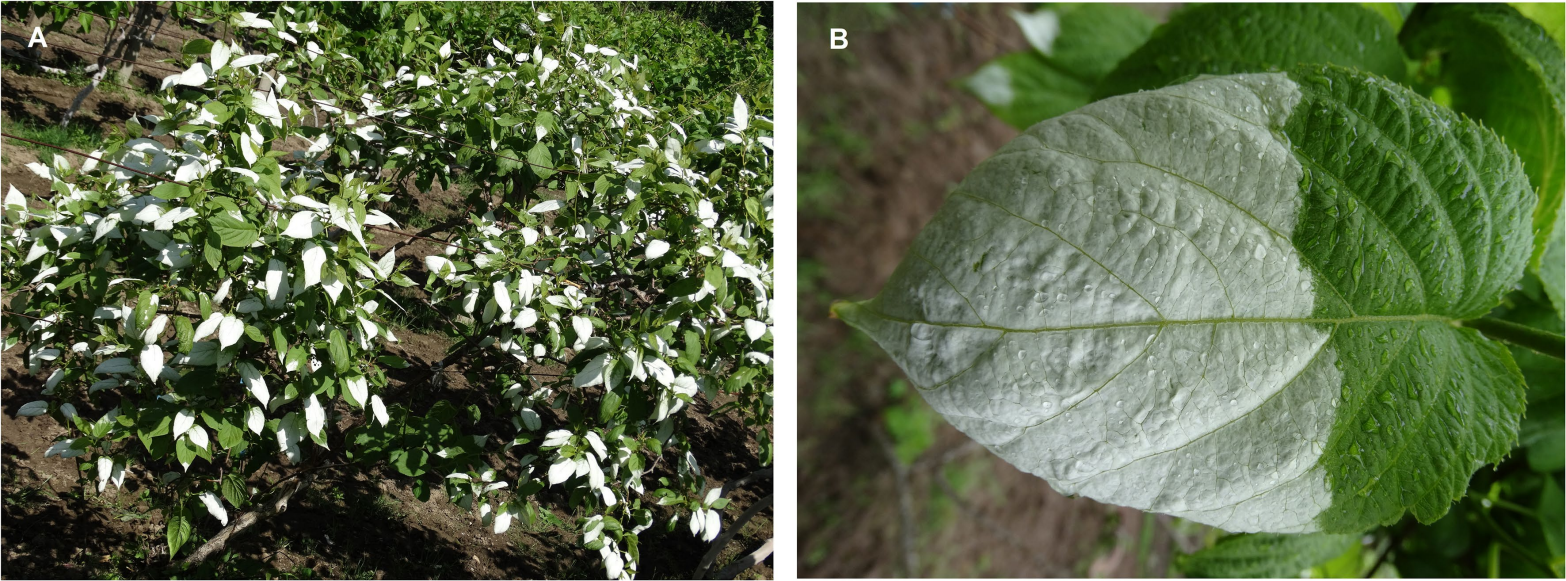
White leaf on reproductive branches in *A. kolomikta* at flower bud stage **(A)** and display of white leaf **(B)**.

*Actinidia kolomikta* (Rupr. & Maxim.) Maxim. is an important germplasm resource for *Actinidia* in China. The species is predominantly distributed in southwestern alpine and northeastern China, the Russian Far East, Eastern Europe, the Korean Peninsula, and Japan. A distinctive feature of the species is that the reproductive branches bear a large number of white leaves (white leaves are lacking on vegetative shoots; Figure 1), and the maximum number of white leaves is attained during flowering. The plant reproduction process (flowering, fruiting, and seed set) is energy and nutrient demanding (Wenk and Falster, 2015). Usually, leaves on reproductive branches appear to provide sufficient photoassimilate to flowers through maintenance of photosynthesis (Lei et al., 2015). However, a low rate of photosynthesis may limit blooming and reproduction. Therefore, we hypothesized that white leaves in *A. kolomikta* may maintain photosynthetic capacity by adjustments of leaf anatomy and physiological regulation. In this study, gas exchange, leaf anatomy, chlorophyll fluorescence, and the transcriptome were examined in white leaves to test the hypothesis.

## Materials and Methods

### Plant Materials

Vegetatively propagated *A. kolomikta* vines were used for experiments at Changchun, China (43°48′45″ N, 125°24′15″ E). The study site receives annual precipitation of 617 mm, and the average annual maximum and minimum temperatures are 37°C and −35°C, respectively. The plants were exposed to full sunlight (the maximum photosynthetic photon flux density [PPFD] on a clear day is ~1,200 ± 128 μmol m^−2^ s^−1^). The maximum light intensity showed no significant change during leaf development. All field trials were conducted from May to July from 2019 to 2021. The mean humidity during the experimental period was 85% at night and 60% in the daytime. Each field plot was divided into three subplots, and the seedlings were planted in each plot (3 m × 4 m). The nitrogen (30.65 ± 2.06 g/Kg), phosphorous (3.44 ± 0.26 g/Kg) and potassium (3.48 ± 0.26 g/Kg) concentrations were sufficient. Seedlings in all experiments were of uniform size. White and green leaves were sampled during late May and early June. Investigation trials were conducted at Changchun and in natural habitats.

### Gas Exchange Measurement

Net photosynthetic rate (*P*_n_), stomatal conductance (*G*_s_), and intercellular CO_2_ concentration (*C*_i_) were measured under ambient conditions with a portable photosynthesis system (CIRAS-2; PP-Systems, Hitchin, UK). The photosynthesis parameters were recorded at 2-hour intervals between 05:00 and 17:00 on sunny days.

Data for the photosynthetic light response curve were recorded from three white leaves between 08:00 and 11:00 (to avoid midday depression of photosynthesis). Measurements were recorded separately on the adaxial and abaxial leaf surfaces. The PPFD was gradually decreased stepwise using an integrated light-emitting diode (LED) light source and ranged from 1,200 to 0 μmol m^−2^ s^−1^. The net photosynthetic rate (*P*_n_) at each PPFD was recorded when it was stable (usually 2–3 min). Quantum use efficiency (apparent quantum yield, AQY) was calculated according to the initial slope of the linear (light-limited) portion of the light-response curve between PPFD of 0 and 200 μmol m^−2^ s^−1^. Initial carboxylation efficiency (CE) was calculated according to the initial slope of the linear (light-limited) portion of the CO_2_-response curve between CO_2_ of 0 and 200 mmol mol^−1^.

### Observation of Leaf Anatomy and Chloroplast Ultrastructure

To understand the type of white leaf and green leaf, leaf structure and ultrastructure were observed using light microscopy and transmission electron microscopy (TEM). Sample preparation for semi-thin sections and TEM was performed in accordance with previously described methods (Konoplyova et al., 2008; Sheue et al., 2012). Semi-thin sections were stained with 1% toluidine blue and observed under a light microscope (ECLIPSE 80i, Nikon Corporation, Tokyo, Japan; Sheue et al., 2012; Wang et al., 2015). Photomicrographs were captured using a Zeiss Axiolab with a digital camera (DXM1200, Nikon). Ultrathin sections were observed using a TEM (JEM-1200EX; JEOL Ltd., Tokyo, Japan) at 80 kV. Leaf thickness parameters were measured using an ocular micrometer and measurements were rounded to the nearest 0.1 μm.

### Measurement of Chlorophyll *a* Fluorescence

During leaf development, leaves were sampled at pre-dawn (03:00–05:00). Chlorophyll *a* fluorescence transience (OJIP) was measured with a Plant Efficiency Analyzer (Pocket-PEA, Hansatech Instruments, King’s Lynn, United Kingdom). Saturating red light of 3,000 μmol m^−2^ s^−1^ was produced by an array of four LEDs (peak wavelength 650 nm). Fluorescent signals were recorded within a time scan from 10 μs to 1 s with a data acquisition rate of 100 readings ms^−1^ for the first 2 ms, and 1 reading ms^−1^ after 2 ms. The maximum quantum yield of PSII (*F*_v_/*F*_m_) was calculated in accordance with the method of Strasser (1997). The *F*_v_/*F*_m_ was determined using a 1 s pulse of red radiation (3,500 μmol m^−2^ s^−1^) on fully dark-adapted (for 1 h) samples. The following parameters from the raw fluorescence measurements were calculated: minimal fluorescence of the dark-adapted state (*F*_0_); the maximal fluorescence of the dark-adapted state (*F*_m_); and variable total chlorophyll fluorescence yield (*F*_v_), defined as *F*_m_ − *F*_0_. Quantum yield for electron transport, ET_0_/ABS = [1–(F_0_/Fm)] × (ET_0_/TR_0_). The efficiency of electron flow beyond QA^–^, Ψ_0_ = 1–V_J_.

Autofluorescence imaging and determination of *F*_v_/*F*_m_ were performed in accordance with previously described methods (Jacobs et al., 2016). Cross sections were prepared from dark-adapted leaves with a Plant Microtome (NK SYSTEM, Japan). Fluorescence images were captured with a fluorescence kinetic microscope equipped with FluorCam7 software (Photon Systems Instruments, Czech Republic). Initial fluorescence (*F*_0_) was recorded in leaves adapted to the dark for 1 h. A 0.8 s pulse of saturating white light (>4,000 μmol m^−2^ s^−1^) was applied to determine the maximum fluorescence (*F*_m_). The *F*_v_/*F*_m_ was calculated following the procedure of Jacobs et al. (2016).

### RNA Sequencing and Differential Gene Expression Analysis

Total RNA was extracted in triplicate from white leaves and green leaves (the control). Illumina paired-end read libraries were constructed using the NEBNext^®^ Ultra RNA Library Prep Kit for Illumina (New England Biolabs, Ipswich, MA, United Sates), and sequenced using an Illumina HiSeq 2000 platform (GENEWIZ, Inc.). Trimming was done to obtain ≥50 million paired reads per sample. The remaining clean reads were assembled using Trinity, which is a novel method for efficient and robust de novo reconstruction of the transcriptome from RNA sequencing (RNA-seq) data. Gene expression levels were calculated using the reads by kilobase per million (RPKM) method. Differentially expressed genes (DEGs) were identified using the false discovery rate (FDR) procedure (Robinson et al., 2010). Selection of DEGs was based on two criteria: (i) FDR ≤ 0.05, and (ii) fold change ≥ 2.0 for green leaves vs white leaves. Significantly enriched gene ontology (GO) terms were determined using GOseq using a hypergeometric test. We applied *p* < 0.05 as the cutoff for a significantly enriched GO term. The Kyoto Encyclopedia of Genes and Genomes (KEGG) is a collection of databases for genomes, biological pathways, diseases, drugs, and chemical substances^1^. We used in-house scripts to identify significantly enriched DEGs in KEGG pathways. The criterion *Q* ≤ 0.05 was applied for significantly enriched KEGG pathways.

To validate the reliability of RNA-Seq, qRT-PCR for transcripts was carried out as described by Sun et al. (2019). qRT-PCR was performed using the SYBR Premix Ex Taq^TM^II (TaKaRa, Bio., Dalian, China) according to the manufacturer’s protocol. The relative expression levels of the selected genes were calculated using the 2^−ΔΔct^ method (Zhang et al., 2022). The sequences of the primers used for qRT-PCR are listed in Supplementary Table S1.

### Statistical Analysis

Statistical analysis was performed using JMP 6.0 software (SAS Institute, Cary, NC, United States). The variables analyzed were as follows. Gas exchange data reported are the mean of at least individual measurements of five leaves. The photosynthetic light response curve data represent the means of individual measurements of three leaves. Chl a fluorescent parameters represent means individual measurements of eight leaves. Autofluorescence imaging were calculated and are presented as the average of five independent measurements. qRT-PCR is presented as the average of three independent measurements.

## Results

### Gas Exchange in White Leaves

**Figure 2:**
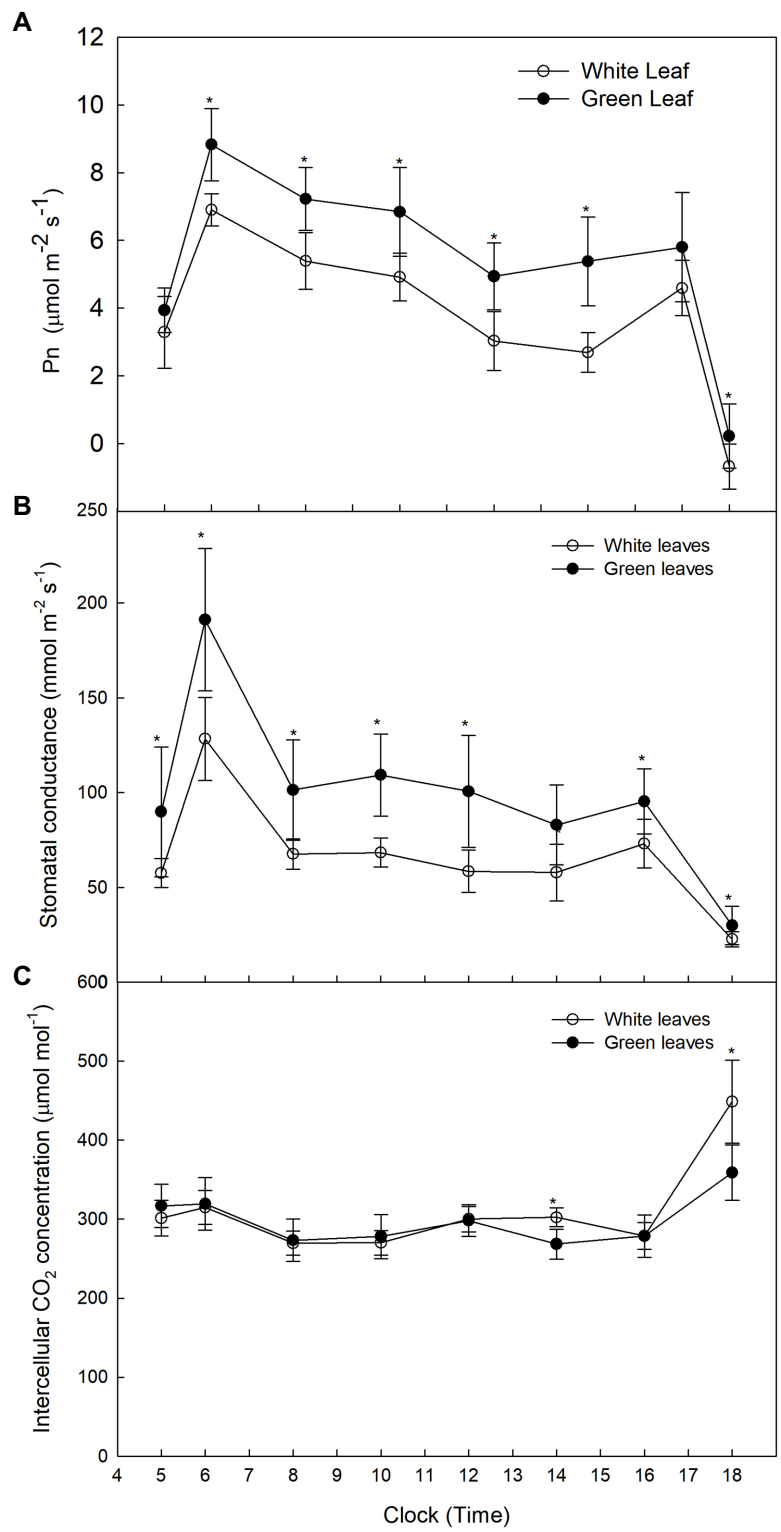
Diurnal variation of *P*_n_ in *A. kolomikta* green leaves and white leaves C: Diurnal variation of *P*_n_ **(A)**, Diurnal variation of *G*_S_ **(B)**, and Diurnal variation of *C*_i_ **(C)**.

Diurnal variation of *P*_n_ in green leaves exhibited a double-peak pattern with peaks observed at 09:15 and 16:45 (Figure 2A). The diurnal trend of *P*_n_ in white leaves was similar to that observed in green leaves (Figure 2A). Although *P*_n_ in green leaves was higher than that of white leaves, the value of *P*_n_ in white leaves was 82% of that in green leaves at 09:15 (Figure 2A). The diurnal trend of *G*_S_ in green leaves and white leaves also exhibited a double-peak pattern, but the *G*_S_ of green leaves was higher than that of white leaves (Figure 2B). Diurnal variation of *C*_i_ in green leaves and white leaves showed almost no significant difference (Figure 2C).

**Figure 3:**
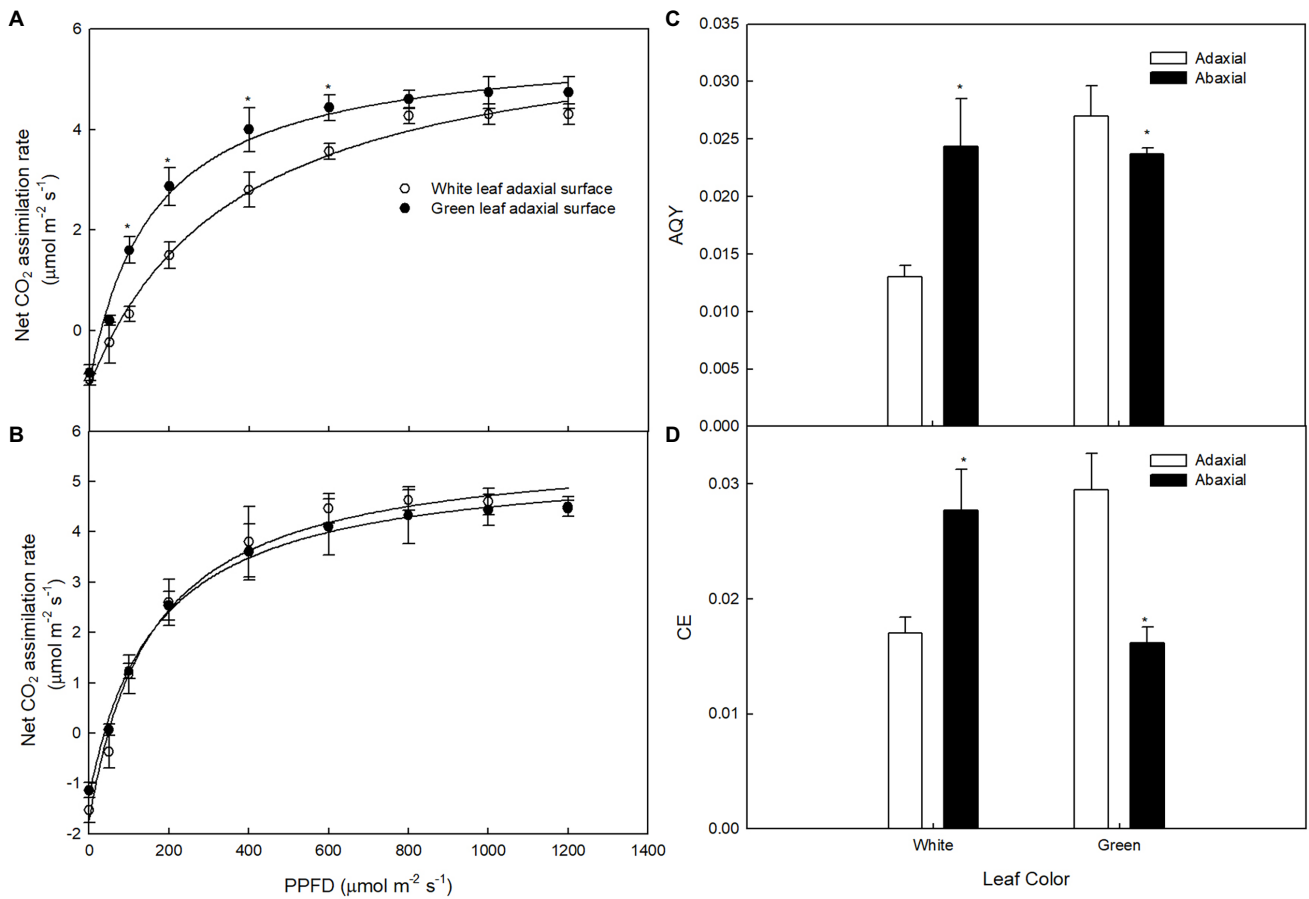
The responses of photosynthesis (*P*_n_) to photosynthetic photon flux density (PPFD) in white and green leaves *P*_n_ to PPFD on adaxial surfaces of white and green leaves **(A)**, *P*_n_ to PPFD on abaxial surfaces of white and green leaves **(B)**, AQY on adaxial and abaxial surfaces of white and green leaves **(C)**, CE on adaxial and abaxial surfaces of white and green leaves **(D)** Mean ± SE of three replicates are presented.

To exclude the impact of light intensity on photosynthesis, *P*_n_ under different PPFDs was measured on the adaxial and abaxial leaf surfaces. The light-saturated *P*_n_ of green leaves was higher than that of white leaves measured on the adaxial surface (Figure 3A), but little difference in light-saturated *P*_n_ values between green and white leaves was observed when measured on the abaxial surface (Figure 3B). The AQY and CE of photosynthesis of white leaves was 51.8 and 41.2% lower than that of green leaves on the adaxial surface (Figures 3C,D). The *P*_n_ and AQY of green and white leaves showed no significant differences when measured on the abaxial surface (Figure 3C), but CE of white leaves was 72% higher than that of green leaves on the abaxial surface (Figure 3D).

### Leaf Anatomy and Ultrastructure

**Figure 4:**
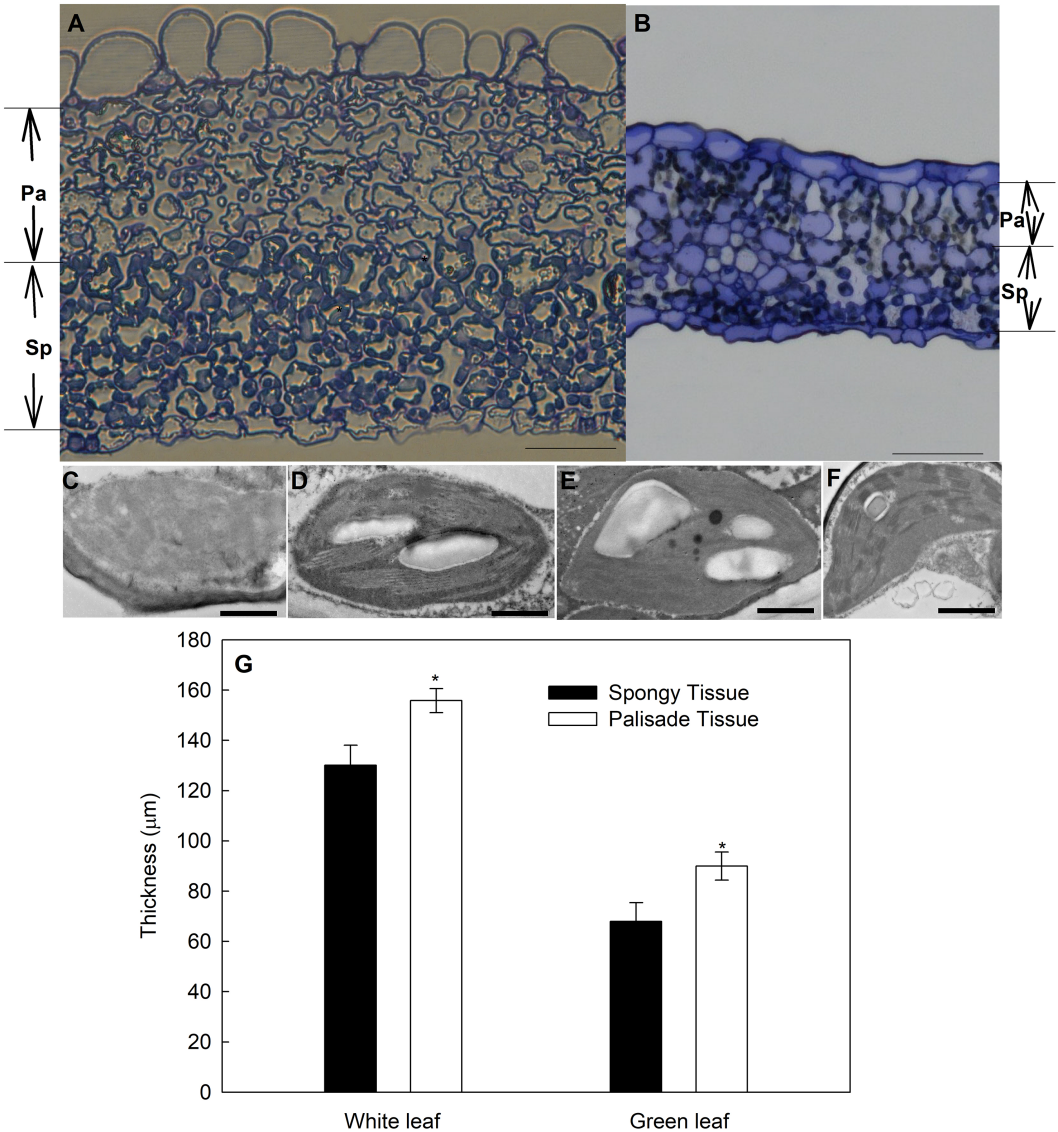
Anatomical features of green and variegated leaves of variegated *A. kolomikta* in late-May when variegated leaves were white. **(A)** Transverse sections from variegated leaves. **(B)** Transverse sections from green leaves. Chloroplast of palisade tissue **(C,E)** and spongy tissue **(D,F)** in white and Green leaves. (**G**) Mean thickness of different tissues in green and white leaves of *A. kolomikta.* Pa, palisade tissue; Sp, spongy tissue. Scale bars: **(A,B)** = 50 μm, **(C–F)** = 500 nm.

The anatomy of green leaves and white ones differed distinctly (Figure 4). The palisade mesophyll of green leaves consisted of a well-established layer of packed and elongated cylindrical cells (Figure 4B) that were in tight contact with the adaxial epidermis. However, in white leaves, only rounded or irregularly shaped cells were observed (Figure 4A), rather than the typical palisade-type cells. Intercellular spaces were observed between the epidermal and mesophyll cells or within the mesophyll cell layer in white leaves (Figure 4A). In cross section, the palisade and spongy mesophyll tissues of white leaves were thicker than those of green leaves (Figure 4G). Compared with green leaves, the thickness of the palisade and spongy mesophyll of white leaves increased by approximately 73% and 91%, respectively.

Observation of the chloroplast ultrastructure by TEM revealed that palisade and spongy parenchyma cells of green leaves contained normal chloroplasts with abundant thylakoid membranes and starch grains (Figures 4E,F). The number of starch grains in palisade parenchymal cells was less than that in spongy parenchymal cells (Figures 4E,F). In contrast, chloroplast development of palisade parenchyma cells in white leaves was abnormal and impaired, and some chloroplast membranes were absent or appeared faint (Figure 4C). Compared with palisade parenchyma cells of white leaves, spongy parenchymal cells of white leaves contained regular and functional chloroplasts with abundant grana and obvious starch grains (Figure 4D).

### Chlorophyll Fluorescence Characteristics

**Figure 5:**
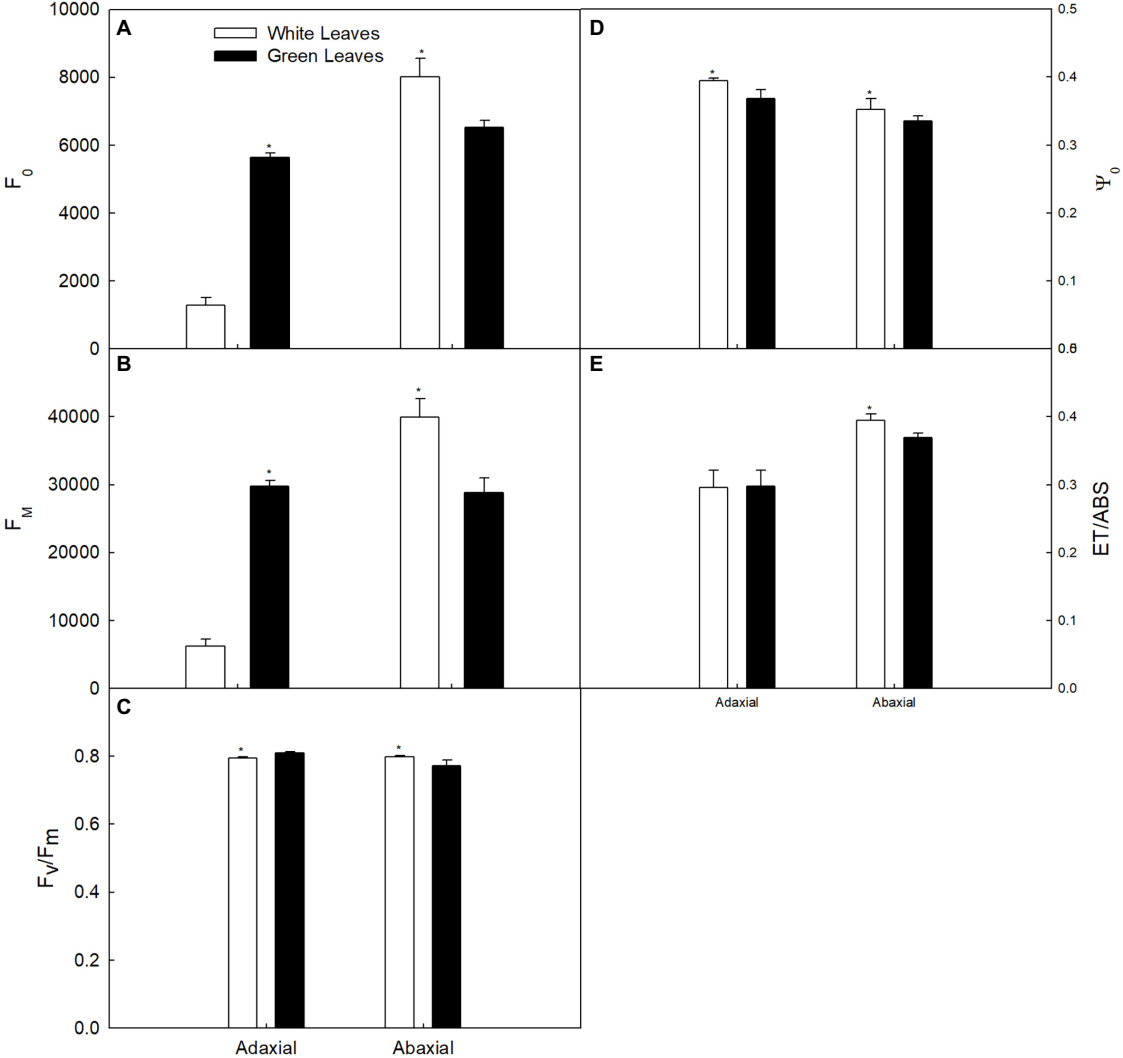
Changes in the chlorophyll fluorescence parameter in white and green leaves. **(A)** *F*_0_. **(B)** *F*_m_. **(C)** The maximum quantum yield of photosystem II (*F*_v_/*F*_m_). **(D)** *F*_0_. **(E)** *F*_0_.

The values of *F*_0_ and *F*_m_ of green leaves were about 4.4-fold and 4.8-fold higher, respectively, compared with those of white leaves measured on the adaxial surface (Figures 5A,B). However, *F*_0_ and *F*_m_ in green leaves measured on the abaxial surface were about 81% and 72%, respectively, of those observed in white leaves (Figures 5A,B). The *F*_v_/*F*_m_ of green leaves was higher than that of white leaves when measured on the adaxial surface, whereas *F*_v_/*F*_m_ in green leaves was lower than that of white leaves when measured on the abaxial surface (Figure 5C). Efficiency of electron flow beyond QA^–^(Ψ_0_) of white leaves was significantly higher than in green leaves (Figure 5D). Quantum yield for electron transport (ET_0_/ABS) of white leaves on the adaxial surface was similar to green leaves, but on the abaxial surface, ET_0_/ABS of white leaves was significantly higher than in green leaves (Figure 5E).

**Figure 6:**
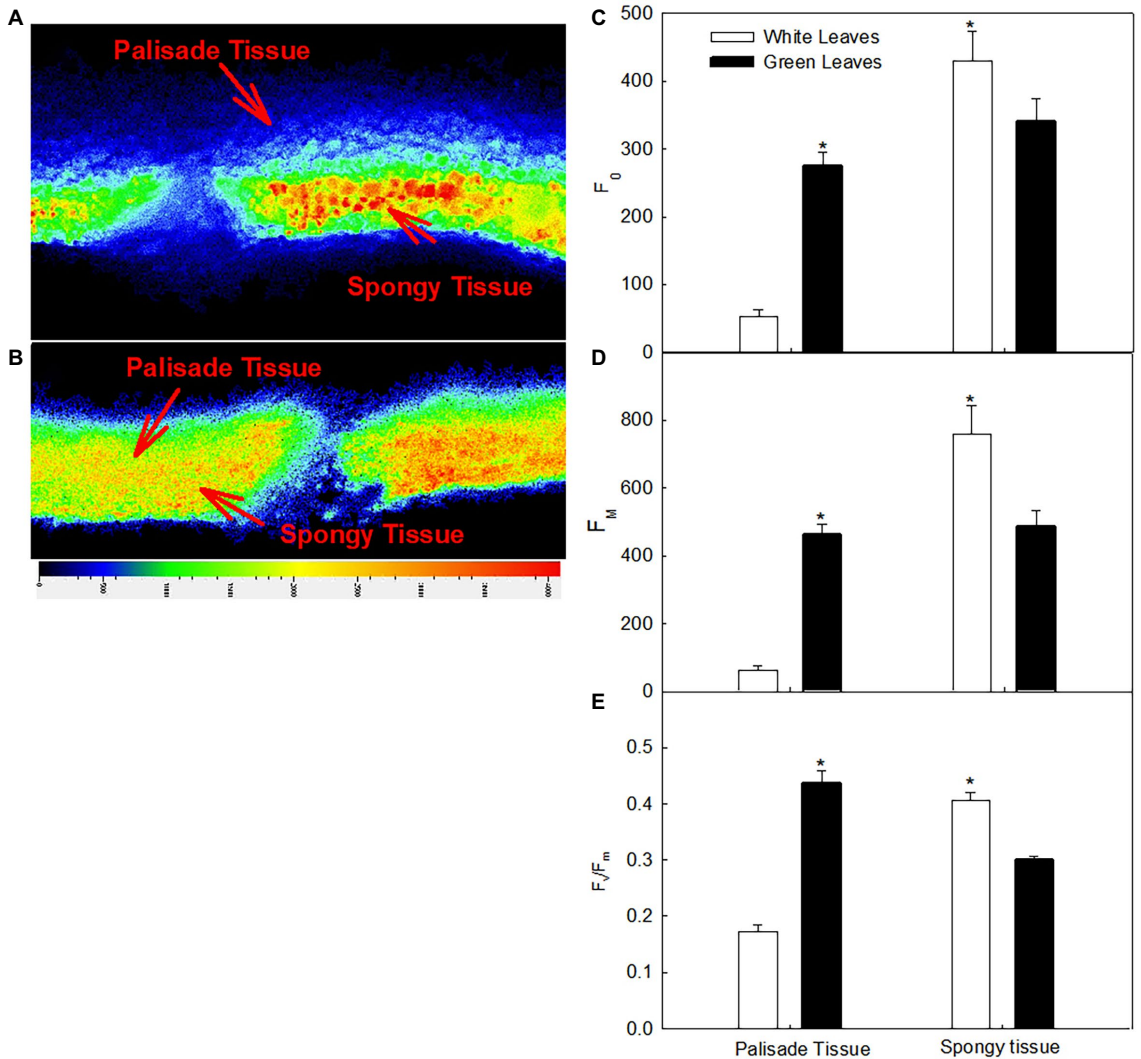
Changes in the chlorophyll fluorescence images and quantum yield of different tissues in white **(A)** and green leaves **(B)**; *F*_0_ (C), *F*_m_ **(D)**, and *F*_v_/*F*_m_ **(E)** of different tissues in white and green leaves. Data represent means (*n* = 5) ± SE.

Direct insight into the photosynthetic efficiency of different mesophyll tissues in leaf cross section can be gained by chlorophyll fluorescence imaging (Figures 6A,B). The fluorescence intensity of the palisade mesophyll in green leaves was significantly higher than that of the spongy mesophyll (Figure 6B), whereas the fluorescence intensity of the palisade mesophyll in white leaves was significantly lower than that of the spongy mesophyll (Figure 6A). The values of *F*_0_ and *F*_m_ of the palisade mesophyll in green leaves were approximately 5.3-fold and 7.4-fold higher, respectively, than those of white leaves (Figures 6C,D). However, *F*_0_ and *F*_m_ of the spongy mesophyll in green leaves was 79% and 64%, respectively, of that of white leaves (Figures 6C,D).

The *F*_v_/*F*_m_ of the different mesophyll tissues in green leaves and white leaves differed significantly. The *F*_v_/*F*_m_ of the palisade mesophyll in green leaves was 136% higher (*p* < 0.05) than that of white leaves (Figure 6E), whereas the *F*_v_/*F*_m_ of the spongy mesophyll of green leaves was 68% of that of white leaves (Figure 6E).

### Transcriptome Analysis

**Figure 7:**
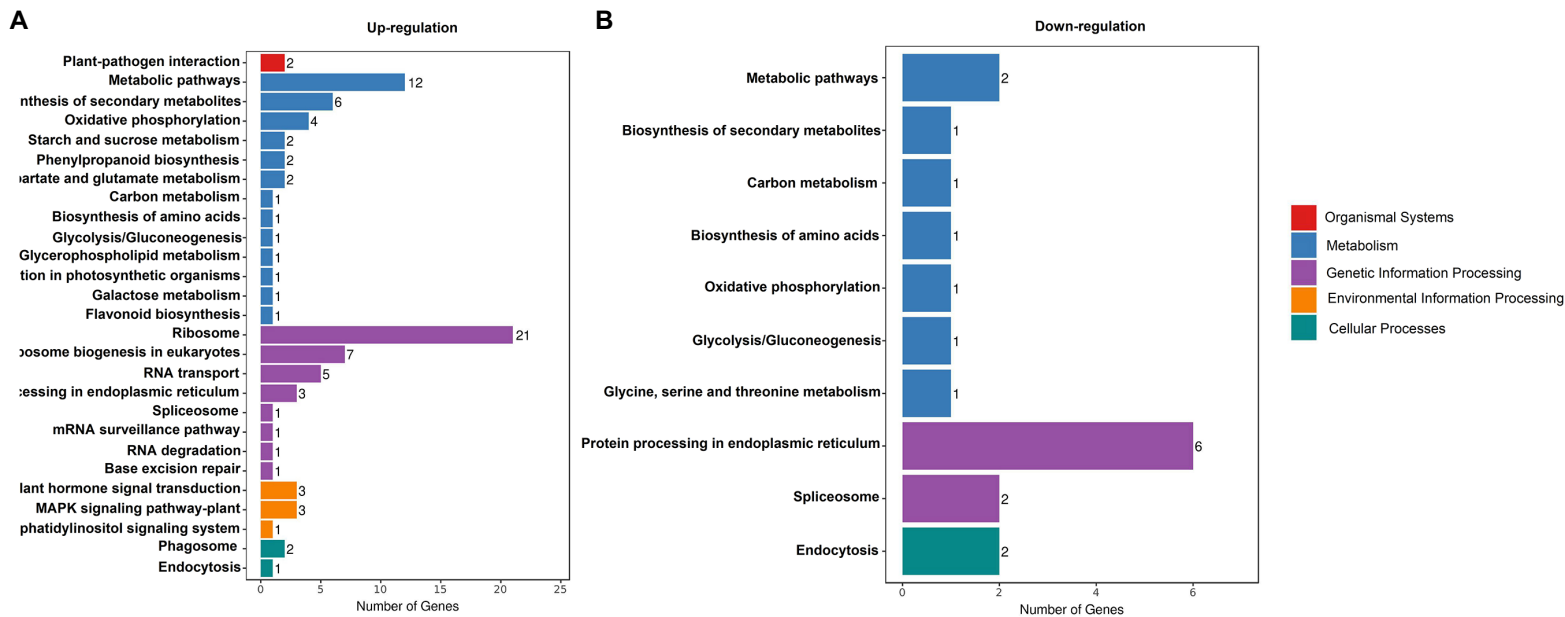
KEGG Classification. Enriched the Kyoto Encyclopedia of Genes and Genomes (KEGG) terms (biological process) up- **(A)** and downregulated **(B)** genes in white leaves compared to green leaves.

To gain insight into changes in expression of photosynthesis-associated genes, a comprehensive transcriptome analysis comparing white leaves and green leaves at flowering was conducted. The RNA-seq analysis identified 501 DEGs (*q*-value <0.001). Compared with green leaves, 215 and 286 genes were exclusively upregulated and downregulated, respectively, in white leaves. To further investigate the biochemical pathways in which these DEGs participate, we analyzed all DEGs according to terms in the KEGG database. 105 genes had KEGG annotations and were categorized into 28 pathways (Figure 7). Significant enrichment of DEGs associated with organismal systems, metabolism, genetic information processing, environmental information processing, and cellular processes was observed. In particular, three pathways were significantly enriched: genes involved in carbon metabolism, starch and sucrose metabolism, and carbon fixation in photosynthetic organisms were significantly upregulated (Figure 7).

To detect genes associated with photosynthesis, we identified seven DEGs that had been previously associated with photosynthetic electron transport and carbon assimilation (Table 1). These genes encode a photosystem II cp43 protein (*CP43*), Chloroplast Nucleoids DNA-binding Protease (*CND41*), NAD(P)H-quinone oxidoreductase subunit 5 (*NDHF*), glyeraldehyde-3-phosphate dehydrogenase (*GAPDH*), RuBisCO activase (*RCA*), and sucrose-phosphate synthase (*SPS*). Some important genes involving photosynthesis were upregulated in white leaves, for example, *GAPDH*, *RCA*, and *SPS*.

**Table 1:** Expression of photosynthesis-related genes in white leaf comparing to green leaf.

Transcript	Symbol	Annotation	logFC	UP/DOWN
comp137243_c1_seq7	*CP43*	Photosystem II cp43 protein	3.42	DOWN
comp143513_c0_seq1	*CND41*	Chloroplast Nucleoids DNA-binding Protease	8.73	DOWN
comp136312_c0_seq3	*NDHF*	NAD(P)H-quinone oxidoreductase subunit 5	7.87	DOWN
comp120940_c4_seq1	GAPDH	Glyceraldehyde-3-phosphate dehydrogenase	3.94	UP
comp138123_c1_seq2	*RCA*	Ribulose bisphosphate carboxylase/oxygenase activase 2	7.11	UP
comp128464_c0_seq1	*CND41*	Chloroplast Nucleoids DNA-binding Protease	2.54	DOWN
comp131156_c0_seq51	*SPS*	Sucrose-phosphate synthase	5	UP

### Real-Time PCR Validation of Differential Gene Expression

**Figure 8:**
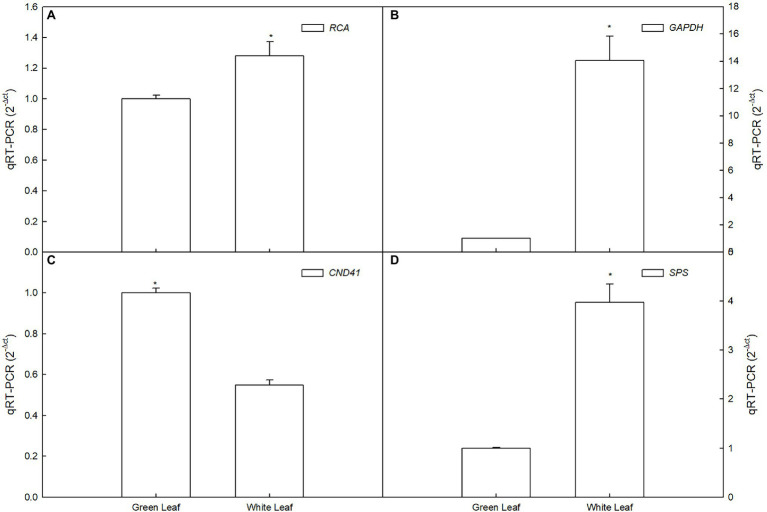
Expression analyses of RCA, GAPDH, CND41, and SPS.

To confirm the RNA-Seq data, we quantified 3 transcripts by qRT-PCR between white leaf and green leaf, which mainly include sucrose metabolism, photosynthesis, and antioxidant-related genes. The expression of *GAPDH*, *RCA*, and *SPS* of white leaf was 14-, 1.3-, and 3.9-fold higher than green leaf respectively (Figures 8A,B,D), the expression of *CND41* of green leaf was 1.8-fold higher than white leaf (Figure 8C). These results from the real-time PCR matched the RNA Seq data very well, thus confirming the expression pattern of those genes in white leaf and green leaf (Figure 8).

## Discussion

### Photosynthesis of White Leaves Supporting Flowering

Plants with non-green leaves (such as white or red leaves) show a significant reduction of photosynthetic capacity compared with that of plants with green leaves (Burger and Edwards, 1996; Gould et al., 2002; Hughes et al., 2005; Zeliou et al., 2009). In some cases, photosynthetic function was almost completely lost in non-green leaves of *Arabidopsis* (e.g., the *Var* variegation; Yu et al., 2011; Lundquist et al., 2014) and *Ficus pumila* “Sonny” (Sheue et al., 2012). It is proved that some specific ecological functions of non-green leaves are usually at the expense of photosynthesis. For example, white bracts of *Davidia involucrata* and *Saururus chinensis* maintained an extremely low *P*_n_, although the plants attracted significantly higher numbers of pollinators (Karageorgou et al., 2008; Sun et al., 2008; Zhao et al., 2013; Song et al., 2018). It is reported that approximately 2,500–3,000 proteins and enzymes are involved in the photosynthetic process (Motohashi et al., 2003; Eberhard et al., 2008; Ouyang et al., 2011), and expression of the genes encoding these proteins and enzymes is correlated with photosynthetic capacity (Müller et al., 2010). In the present study, we detected only seven DEGs associated with photosynthetic electron transport and carbon assimilation (Table 1). This finding implied that the majority of photosynthesis-associated genes showed no significant difference in expression between white leaves and green ones.

More importantly, the present study demonstrated that the white leaves of *A. kolomikta* still maintained a relatively high *P*_n_ (Figures 2, 3), and the highest *P*_n_ ratio of white leaves to green ones was 82% over the course of the day (Figure 2A). In addition, the total *P*_n_ of white leaves over the course of the day was 70% of that of green ones. Notably, white leaves comprise almost 42–57% of the total leaf number during blossoming. Therefore, the photoassimilates from white leaves on a reproductive branch might effectively support flower development on that branch.

### Mechanism for Maintenance of Photosynthesis in White Leaves

The present results also demonstrated that the *P*_n_ of white leaves and green leaves under different PPFDs differed on the adaxial leaf surface (Figure 3). Although the *P*_n_ of white leaves and green ones under saturated light showed little difference, the *P*_n_ of green leaves was significantly higher than that of white leaves under low light intensity. Thus, the AQY of green leaves was significantly higher than that of white ones on the adaxial leaf surface (Figure 3A). Significant decreases of *G*_s_ in white leaves were associated with changes in *C*_i_ (Figures 3B,C), which suggested that the reduction of *P*_n_ in white leaves was caused by non-stomatal limitation.

Leaf structure is an important determinant of leaf photosynthetic characteristics (Green and Kruger, 2001). However, contrary to previous studies, the present results showed that functional chloroplasts were not developed in palisade parenchyma cells (Figure 4C), although the palisade mesophyll of white leaves was significantly thicker than that of green ones (Figure 4G). In contrast, functional chloroplasts and higher abundance of starch grains were observed in the spongy mesophyll cells of white leaves (Figure 4D). These structural traits suggested that photosynthetic activity of the spongy mesophyll in white leaves might be greater than that of the palisade mesophyll. Compared with green leaves, although the fluorescence intensity and *F*_v_/*F*_m_ on the adaxial surface of white leaves were significant lower (Figure 5C), Ψ_0_ and ET_0_/ABS were significant higher or similarity. Furthermore, these parameters measured on the abaxial surface of white leaves were significantly higher than those of green ones (Figure 5C). Photochemical activity on the adaxial and abaxial sides of leaves mainly reflect photosynthetic activity of the palisade and spongy mesophyll tissues (Peguero-Pina et al., 2009; Wu et al., 2020). Therefore, higher fluorescence intensity, *F*_v_/*F*_m_ and quantum yield for electron transport on the abaxial side of white leaves (Figure 5) demonstrated indirectly that the photosynthetic apparatus and photochemical activity of the spongy mesophyll in white leaves were enhanced. The data from fluorescence kinetic microscope with leaf cross section further confirm that the fluorescence intensity and *F*_v_/*F*_m_ of the spongy mesophyll in white leaves were significantly higher than those of green ones (Figure 6). These results reflected that the spongy mesophyll of white leaves may compensate the constraints to the photosynthetic apparatus and photochemical activity in the palisade mesophyll. The structural traits and photochemical activity in white leaves may explain that the photosynthetic capacity of white leaves and green ones showed little difference when the *P*_n_ under different PPFDs was measured on the abaxial leaf surface. These results showed that the photosynthetic apparatus is located predominantly in the palisade mesophyll in green leaves, whereas in white leaves the spongy mesophyll accumulated more components of the photosynthetic apparatus. In addition, the lower AQY and CE on the adaxial side of white leaves might be due to higher light reflection and abnormal chloroplast development in palisade mesophyll cells of white leaves, which resulted in a lower light energy utilization ratio. However, AQY and CE on the abaxial side of white leaves was slightly higher than that in green leaves (Figure 3C). These data further suggested that the higher light energy utilization ratio of the spongy mesophyll facilitated maintenance of the photosynthetic capacity of white leaves. Therefore, changes of the leaf structure in white leaves may greatly influence the photochemical activity and carbon assimilation capacity of different cell layers.

Previous studies suggest that white areas of *Begonia formosana* leaves can maintain higher photosynthetic capacity (Sheue et al., 2012), which is the result of palisade mesophyll cells in white areas containing functional chloroplasts. In addition, in some plants, for example, *Ranunculus ficaria* and *Arum italicum* (Konoplyova et al., 2008; Rocca et al., 2011), white leaves possess two or three palisade cell layers, which effectively maintain the photosynthetic capacity of white leaves. However, for *A. kolomikta*, the specialized structure of white leaves suggested that the spongy mesophyll compensated for lower photosynthetic capacity of the palisade mesophyll and that the functioning of the spongy mesophyll as the main photosynthetic tissue plays a vital role in maintenance of the photosynthetic capacity of white leaves. Thus, the photosynthetic mechanisms of white leaves in *A. kolomikta* differ significantly from those of white leaves in other plant.

### Photosynthesis-Related Genes Are Involved in Regulation of Photosynthetic Capacity

Based on the analysis of DEGs between white leaves and green ones, seven of the eight photosynthesis-related DEGs were downregulated (Table 1). These genes regulate expression of proteins or enzymes involved in photosynthetic electron transport and carbon assimilation. Therefore, these downregulated genes may be associated with a slight decrease in photosynthetic capacity of white leaves. *GAPDH* and *SPS* are two key enzymes involving the Calvin–Benson cycle and sucrose biosynthesis (Sun et al., 2022). Rubisco activase (*RCA*) can keep catalytic active state of Rubisco (Zhang et al., 2022). Chloroplast Nucleoids DNA-binding Protease (*CND41*) is involved in Rubisco degradation (Kato et al., 2005). In this study, *RCA* and *GAPDH* gene was upregulated (Table 1 and Figures 8A,B), which suggested higher Rubisco activity and CO_2_ fixation efficiency. Higher expression of *RCA* and *SPS* may match higher CE and obvious starch grains on the abaxial side of white leaves. Upregulation of *SPS* promoted accumulation of starch and sucrose (Figure 8D). Meanwhile, downregulation of *CND41* can avoid Rubisco degradation (Figure 8C). This regulation of transcriptional level may compensate for the lowered photosynthetic capacity of the palisade mesophyll, which could be important in the maintenance of photosynthetic capacity in white leaf.

## Conclusion

Structural characteristics of white leaves containing thicker palisade and spongy mesophyll layer and functional chloroplasts in spongy tissue cell supported the view that spongy tissue as main photosynthetic tissue played an important role in maintaining photosynthetic capacity. Ulteriorly, higher AQY, CE, and photochemical activity (*F*_v_/*F*_m_, Ψ_0_, and ET_0_/ABS) in spongy parenchyma cells correlated positively with structural characteristics and demonstrated furtherly our view. Identification and expression analysis of key DEGs associated with carbon assimilation suggest well-coordinated carboxylation efficiency. Upregulation of *RCA*, *GAPDH*, and *SPS* involved in regulation of Rubisco activase and sugar synthesis may improve photosynthetic capacity of white leaves. Current findings demonstrated the insight that higher photosynthetic capacity of spongy mesophyll may compensate for the photosynthetic losses of the palisade mesophyll.

## Data Availability Statement

The datasets presented in this study can be found in online repositories. The names of the repository/repositories and accession number(s) can be found at: NCBI, accession numbers: PRJNA813327. Further inquiries can be directed to the corresponding authors.

## Author Contributions

Z-xW, and JA designed the research. MY, DS, LC and YY performed the research. G-lS, D-QW, and D-hL analyzed data. Z-xW wrote the paper. All authors contributed to the article and approved the submitted version.

## Funding

This study was supported by the Natural Science Foundation of China (to Z-xW, G-lS, JA, and CDJ, 31870673, 31571576). This work complies with Chinese law.

## Conflict of Interest

The authors declare that the research was conducted in the absence of any commercial or financial relationships that could be construed as a potential conflict of interest.

## Publisher’s Note

All claims expressed in this article are solely those of the authors and do not necessarily represent those of their affiliated organizations, or those of the publisher, the editors and the reviewers. Any product that may be evaluated in this article, or claim that may be made by its manufacturer, is not guaranteed or endorsed by the publisher.

## Footnotes

^1^ http://en.wikipedia.org/wiki/KEGG

## Supplementary Material

Supplementary Table S1 List of primers used in this study.
