# Supplementary material for: Enhancement of Photosynthetic Capacity in Spongy Mesophyll Cells in White Leaves of Actinidia kolomikta
Source: Front Plant Sci. 2022 May 11;13:856732. doi: 10.3389/fpls.2022.856732 (PMC9131848; doi:10.3389/fpls.2022.856732)
Supplement: Supplementary Table S1 — List of primers used in this study. [file Table_1.doc]

**SUPPLEMENTARY MATERIAL**

The Supplementary Material for this article can be found online at: [https://www.frontiersin.org/articles/10.3389/fpls.2022.856732/full#supplementary-material](https://www.frontiersin.org/articles/10.3389/fpls.2022.856732/full#supplementary-material.).

Supplementary Table S1. List of primers used in this study.

Table S1 List of primers used in this study

| Primer name | Sequence（5’ to 3’） | |
| --- | --- | --- |
| CND41-F | TTCTCCATCGGAAGCACCAA | |
| CND41-R | GTTTACAACGCAATTCGAGATGAGT | |
| GAPDH-F | CCCGAGTAAAGACGCACCTATG | |
| GAPDH-R | CCTCAACAATGCCAAACCTATC | |
| RCA-F | GATTCTACATTGCTCCTGCCTTC | |
| RCA-R | TTTCCCTGACCTTTGCCTCC | |
| SPS-F | AATCGTTAAGGGATGTGGAAGAC | |
| SPS-R | TGGAAGGGCTATTGGAGGAA | |
|  |  |  |
